# A Brief Review of Low-Dose Rate (LDR) and High-Dose Rate (HDR) Brachytherapy Boost for High-Risk Prostate

**DOI:** 10.3389/fonc.2019.01378

**Published:** 2019-12-10

**Authors:** Benjamin W. Fischer-Valuck, Hiram A. Gay, Sagar Patel, Brian C. Baumann, Jeff M. Michalski

**Affiliations:** ^1^Department of Radiation Oncology, Winship Cancer Institute, Emory University, Atlanta, GA, United States; ^2^Department of Radiation Oncology, Washington University School of Medicine in St. Louis, St. Louis, MO, United States

**Keywords:** HDR, LDR, brachytherapy, boost, high risk prostate, cancer

## Abstract

For patients with unfavorable or high-risk prostate cancer, dose escalated radiation therapy leads to improved progression free survival but attempts to deliver increased dose by external beam radiation therapy (EBRT) alone can be limited by late toxicities to nearby genitourinary and gastrointestinal organs at risk. Brachytherapy is a method to deliver dose escalation in conjunction with EBRT with a potentially improved late toxicity profile and improved prostate cancer related outcomes. At least three randomized controlled trials have demonstrated improved biochemical control with the addition of either low-dose rate (LDR) or high-dose rate (HDR) brachytherapy to EBRT, although only ASCENDE-RT compared brachytherapy to dose-escalated EBRT but did report an over 50% improvement in biochemical failure with a LDR boost. Multiple single institution and comparative research series also support the use of a brachytherapy boost in the DE-EBRT era and demonstrate excellent prostate cancer specific outcomes. Despite improved oncologic outcomes with a brachytherapy boost in the high-risk setting, the utilization of both LDR, and HDR brachytherapy use is declining. The acute genitourinary toxicities when brachytherapy boost is combined with EBRT, particularly a LDR boost, are of concern in comparison to EBRT alone. HDR brachytherapy boost has many physical properties inherent to its rapid delivery of a large dose which may reduce acute toxicities and also appeal to the radiobiology of prostate cancer. We herein review the evidence for use of either LDR or HDR brachytherapy boost for high-risk prostate cancer and summarize comparisons between the two treatment modalities.

## Introduction

Nearly 180,000 new cases of prostate cancer are estimated to be diagnosed in 2019 (1, 2). For patients with high-risk prostate cancer, treatment options most often include surgery or a combination of androgen deprivation therapy (ADT) and radiation therapy. External beam radiation therapy (EBRT) is the most common method to deliver radiotherapy for localized prostate cancer.Multiple studies have demonstrated that dose-escalated external beam radiation therapy (DE-EBRT) improves local control, freedom from biochemical failure, freedom from distance metastases, and decreases the need for salvage therapy (3–6). DE-EBRT, however, has also been associated with increases in late genitourinary (GU) and gastrointestinal (GI) toxicities (3). In the NRG Oncology/RTOG 0126 randomized clinical trial, the 5-years rates of both GI and GU late toxicity were increased with dose escalation (3). Brachytherapy is a method to deliver high-dose radiotherapy and escalate the biologically equivalent dose (BED), either as monotherapy or in tandem with EBRT as a boost, which is highly conformal and can often provide sparing of the surrounding organs at risk that is often not achievable with EBRT. Both permanent seed low dose-rate (LDR) or high dose-rate (HDR) brachytherapy provide a highly conformal escalation of dose to the cancer and allow greater sparing of surrounding normal organs than that possible with any type of EBRT (7). The American Society of Clinical Oncology (ASCO)/Cancer Care Ontario (CCO) Joint Guideline Update published in 2017 explicitly states that for patients with high-risk prostate cancer receiving EBRT and androgen deprivation therapy (ADT), brachytherapy boost (either LDR or HDR) should be offered to eligible patients (8). This recommendation is largely based on the ASCENDE-RT trial which demonstrated a significant improvement in the rates of biochemical relapse for patients treated with a brachytherapy boost (9). In ASCENDE-RT, the brachytherapy boost was delivered using a permanent seed low-dose rate (LDR) implant. LDR brachytherapy is a proven method with decades of follow-up and endorsement by numerous expert consensus groups. Another method of brachytherapy, high-dose rate (HDR) brachytherapy, is an alternative to LDR which has many properties that may make it a superior alternative to LDR. In contrast to LDR, HDR is not a permanent implant and generally allows for more consistent dose coverage and relative lower dose to the rectum, bladder, and urethra (7, 10, 11). Both LDR and HDR boost are recommended in the ASCO/CCO Joint Guideline recommendations and the choice between the two is often determined by physician, hospital, patient, and disease characteristics. We herein report on the importance of the brachytherapy boost as well as compare and contrast the use of both LDR and HDR brachytherapy as a boost in high-risk prostate cancer, and summarize future directions using these treatment modalities.

## LDR brachytherapy boost

LDR brachytherapy, commonly referred to as permanent prostate brachytherapy or seed implant, is a type of procedure in which implanted radioactive sources are permanently placed into the prostate. Defined by the International Commission on Radiologic Units and Measurements, LDR brachytherapy is the utilization of a radiation source with a dose-rate of <2 Gy per hour (12). Brachytherapy boost delivered with LDR has been a well-established treatment modality in the treatment of high-risk prostate cancer with numerous studies supporting its use and efficacy (13–15). Sathya et al. (16) conducted a randomized controlled trial comparing EBRT to 40 Gy in 20 fractions plus a temporary LDR brachytherapy boost with iridium-192–35 Gy vs. EBRT alone to 66 Gy in 33 fractions in patients with high-risk prostate cancer. No androgen deprivation therapy was given neoadjuvantly or concurrently in either arm. The primary outcome was biochemical or clinical failure. With a median follow-up of 8.2 years, 29% of patients in the EBRT plus temporary LDR brachytherapy boost arm failed vs. 61% in the EBRT alone arm (hazard ratio, 0.42; *p* = 0.0024) (16, 17). While the EBRT dose used in this study was low compared to modern standards, this trial laid the groundwork and confirmed the principal that brachytherapy in conjunction with moderate dose EBRT resulted in increased rates of biochemical control than that achieved with EBRT alone.

Recently, the highly anticipated results of a large randomized trial comparing the now standard dose-escalated EBRT to EBRT plus LDR brachytherapy boost were published. The ASCENDE-RT trial was a randomized Phase III study comparing EBRT alone (78 Gy/39 fractions) to EBRT (46 Gy/23 fractions) plus an LDR brachytherapy boost (115 Gy using ^125^I) in patients with intermediate or high-risk prostate cancer (9). Both arms included 12 months of androgen deprivation therapy. The trial included 398 patients and demonstrated a statistically significant improvement in biochemical progression-free survival (b-PFS) in favor of the brachytherapy boost arm, with 9-years b-PFS of 83 vs. 62% (18). At a median follow-up of 6.5 years, there was no statistical difference in 7-years overall survival although there was a trend toward improvement with the LDR boost (85.7 vs. 81.5%). Longer follow-up of the trial will be necessary to determine if the addition of the LDR-boost correlates to improved metastasis free, cause-specific, and overall survival with these results anticipated with median follow-up of 13 years. With regards to overall survival, Johnson et al. (19) identified patients from the National Cancer Database (NCDB) with unfavorable prostate cancer who were treated with either DE-EBRT or EBRT with a LDR boost. This study attempted to mirror enrollment criteria of ASCENDE-RT but allowed patients to have received ADT up to 8 months prior to definitive radiation therapy. They found that the LDR boost was associated with improved overall survival (7-years OS 82 vs. 73%; *p* <0.001) (19). The improved survival outcome persisted in multivariable analysis and with propensity score matching, although the study cannot fully account for selection bias in the choice of treatment.

## HDR Boost

HDR differs from LDR in that radiation sources with higher activity are temporarily inserted into the prostate gland using catheter needles and then removed after the prescribed dose has been delivered. The International Commission on Radiologic Units and Measurements defines HDR as a dose delivered at a rate >12 Gy/h, although in actuality this is usually much higher, often in excess of 1 Gy per minute (7, 12). With regards to radiation biology, the degree of dose escalation achievable with HDR brachytherapy, compared to other EBRT techniques and LDR, may be more effective in killing prostate cancer cells (7, 20, 21). The rapid dose delivery seen in HDR is considered to be selectively more damaging to cells with lower alpha/beta ratios, such as prostate cancer and late responding normal tissues (22–24). Radiobiologic models thus support current clinical evidence for equivalent outcomes with either LDR or HDR, with theoretical advantages to HDR brachytherapy (22).

In terms of HDR brachytherapy boost, there does exist at least one randomized trial although it predates DE-EBRT. Hoskin et al. (25) performed a randomized controlled trial of a HDR boost vs. EBRT alone in patients with mostly intermediate and high-risk disease (25). Patients were randomized to receive either EBRT alone (55 Gy/20 fractions) or EBRT (35.75 Gy/13 fractions) plus an HDR boost (17 Gy/2 fractions). Neoadjuvant androgen deprivation therapy was given in 76% of patients at the discretion of the treating physician. Men in the HDR boost cohort had a 31% decrease in the risk of local recurrence, and late genitourinary toxicities were similar in both arms (7, 25). Despite providing randomized evidence, criticisms of this trial include a low EBRT alone radiation dose (55 Gy/20 fractions) compared to current standards of 60 Gy in 20 fractions or 78–80 Gy in standard fractionation. A randomized feasibility study was conducted by Vigneault et al. to assess the ability to randomize patients between dose escalated image-guided radiation therapy (IGRT) (78 Gy/39 fractions or 60 Gy/20 fractions) and IGRT plus HDR brachytherapy boost (37.5 Gy/15 fractions + 15 Gy HDR boost) with good compliance although small numbers (57 patients randomized) (26). Rates of protocol deviations and acute toxicities were low in both arms, but no biochemical control rates are reported as data matures (26).

While no other prospective, randomized comparisons of DE-EBRT and HDR boost exist, multiple single institution reports have demonstrated favorable biochemical control rates similar to those in ASCENDE-RT with better toxicity profiles. Vigneault et al. (27) reported on a cohort of 832 men with intermediate and high-risk disease treated with a range of doses of HDR brachytherapy boost in combination with EBRT and found biochemical control of 95% with median follow-up of 66 months (27). In this trial, they reported that late grade 3 GU toxicity ranged from ~2–5% dependent on the dose level the patient was treated on (27). There were no grade 3 GI toxicities reported. Androgen suppression was used in 41.3% of patients in this study (4–6 months in intermediate cases and 18–36 months in high-risk cases). There was significant differences in the median follow-up between the different HDR dose levels which did not allow for valid comparison of biochemical control rates between the different groups. Martinez et al. reported on a dose escalation trial using a HDR brachytherapy boost and found very favorable 10-years PSA control approaching 81% in men receiving the escalated dose treatment (28). Of the over 470 patients treated with EBRT plus HDR, grade 3 genitourinary toxicity was extremely rare at <1% (28). Neoadjuvant and/or concurrent androgen suppression was used in 51.3% of the patients. Additional data is also emerging in support of an HDR boost in the high-risk setting. Kent et al. (29) recently published results of their single institution retrospective review of 46 Gy EBRT plus HDR boost (median boost 18 Gy/3 fractions) compared to EBRT alone (median 70 Gy). The 5, 10, and 15-years overall survival was higher at 92, 81, and 67%, respectively, for the EBRT plus HDR cohort, compared with 88, 71, and 53%, respectively, in the EBRT alone cohort (*p* <0.001) (29). The 5, 10, and 15-years cause specific survival was also higher in the HDR boost group with survival of 96, 93, and 87% (EBRT plus HDR) and 95, 88, and 79% (EBRT alone), respectively (*p* <0.037) (29). A limitation of this study is the heterogenous use of androgen suppression at the discretion of the treating physician and specifically an increased use of ADT in the EBRT plus HDR group. Also, a median dose of 70 Gy by standard fractionation in the external beam alone group is again lower than the current standard for dose escalated therapy. Numerous other single institution trials also support the use of HDR brachytherapy boost (11, 30–34) (Supplemental Methods).

## HDR Vs. LDR boost

Multiple studies have suggested that when used in the monotherapy setting for more favorable localized prostate cancer, both HDR and LDR brachytherapy have equivalent biochemical progression-free survival outcomes (35, 36). For high-risk patients, the 2017 American Society of Clinical Oncology (ASCO)/Cancer Care Ontario (CCO) brachytherapy guidelines state that men with high-risk prostate cancer should be offered either an LDR or HDR boost if choosing a definitive radiation management approach (8). The recommendation of a brachytherapy boost was largely based on the previously discussed three randomized controlled trials comparing EBRT alone to EBRT plus a brachytherapy boost and demonstrate improved disease free survival with the boost (Table 1). In each of these trials, however, a different modality/type of brachytherapy boost was used. Data comparing LDR and HDR head-to-head are much more limited in the boost setting.

**Table 1:** Randomized controlled trials comparing external beam radiation therapy (EBRT) vs. EBRT plus brachytherapy boost.

**RCT**	**Year**	**Treatment**	**#Patients**	**Primary outcome**	**OS**	**PCSM**	**MFSR**
Sathya^14^	1992–1997	EBRT	53	BCF: 39%	NR	NR	NR
		EBRT + LDR	51	BCF: 71%			
Hoskin^23^	1997–2005	EBRT	111	bDFS: 4.3 y	88%, 7 y	NR	NR
		EBRT + HDR	109	bDFS: 5.1 y	81%, 7 y		
				*p* = 0.04	*p* = 0.2		
Morris^6^	2002–2011	DE-EBRT	200	bDFS: 62%, 9 y	74%, 7 y	5.5%	9%
		EBRT + LDR	198	bDFS: 83%, 9 y	78%, 7 y	3.5%	8.5%
				*p* <0.001	*p* = 0.29	*p* = 0.32	*p* = 0.83

*RCT, randomized controlled trial; OS, overall survival; PCSM, prostate cancer specific mortality; MFSR, metastasis free survival rate; EBRT, external beam radiation therapy; LDR, low dose rate brachytherapy; HDR, high dose rate brachytherapy; BCF, biochemical failure; bDFS, biochemical disease free survival*.

For men with high-risk disease, Kishan et al. (37) reported on the differences in prostate cancer-specific mortality and distant metastasis in prostate cancer patients with high-risk disease treated with either surgery, EBRT with ADT, or EBRT plus either LDR or HDR brachytherapy with ADT in a large multi-institutional cohort (37). Androgen deprivation therapy was given in 89.5% of patients receiving EBRT alone and 92.4% of patients receiving EBRT plus brachytherapy boost (37). The duration of androgen suppression was significantly shorter in the EBRT plus brachytherapy arm (12 vs. 22 months EBRT alone; *p* <0.001) (37). Despite the difference in androgen suppression duration, this study found that among patients with Gleason 9–10 disease, treatment with EBRT plus brachytherapy and ADT was associated with significantly better prostate cancer-specific mortality and longer time to distant metastases compared to surgery or ADT and EBRT alone (37). They performed a cause-specific regression to determine an effect of LDR vs. HDR on clinical outcomes, including both prostate cancer-specific mortality and distant metastasis, and found no difference between the two techniques (37).

King et al. (38) used the National Cancer Database in an attempt to compare LDR vs. HDR boost with regards to overall survival outcomes. In their study, they estimated overall survival in patients with unfavorable prostate cancer treated with dose-escalated EBRT and EBRT followed by LDR boost vs. HDR boost (38). Patients included were diagnosed with NCCN intermediate or high-risk prostate cancer from a time period of 2004–2014. In their analysis of over 120,000 patients, HDR boost was associated with a similar overall survival compared to LDR boost using multivariable analysis [adjusted hazard ratio (AHR), 1.03 (0.96–1.11); *p* = 0.38]. Compared to dose-escalated EBRT, HDR boost was associated with significantly better overall survival [AHR, 1.36 (1.29–1.44); *p* <0.001] (38). Androgen deprivation therapy was given in 40.4% of patients with the HDR boost, 43.1% of patients with the LDR boost, and 49% of patients with DE-EBRT (*p* <0.001) (38).

## Toxicity Concerns

RTOG P-0019 was a phase II study of EBRT combined with LDR brachytherapy boost (45 Gy/25 fractions + 108 Gy ^125^I boost) for intermediate risk prostate cancer with the primary goal to estimate the acute and late Grade 3-5 GU and GI toxicity (39). Short-term androgen suppression up to 6 months was allowed and 27% received ADT. A total of 138 patients from 28 institutions were enrolled on the study with acute toxicity evaluable in 131 patients (39). Acute Grade 3 GU toxicity was recorded in 7.6% of patients without any Grade 4 or 5 events (39). Six months after radiation therapy, ~63% of patients reported a higher International Prostate Symptom Score (IPSS) score compared to baseline (39). The 18-months estimate of both late Grade 3 GU and GI toxicity was 3.3% (39). With longer follow-up, increased rates of Grade 3 or greater GU/GI toxicity were reported, estimated at 15% (95% CI, 8–21%) at 48 months (40). CALGB 99809 was another multi-institutional trial designed to assess the toxicity and feasibility of EBRT plus LDR brachytherapy boost (45 Gy/25 fractions + 100 Gy ^125^I or 90 Gy ^103^Pd boost) combined with 6 months of ADT (41). Acute Grade 2 and 3 toxicity occurred in 25 and 7% of men and was most commonly urinary frequency/urgency (41). Late Grade 2 and 3 toxicity was observed in 20% and 2% of men, respectively (41). Differences between these two multi-institutional protocols included an expansion on the LDR boost clinical target volume (CTV) of 5 mm (0 mm posteriorly) in the RTOG trial compared to no expansion in the CALGB trial, which may contribute to the rates of late Grade 3 or greater toxicities.

In the randomized ASCENDE-RT trial, toxicity was increased in the brachytherapy group with the cumulative incidence of grade 3 GU events at 5 years of ~18% for the brachytherapy boost arm vs. 5% for the EBRT alone arm (*p* <0.001). There was also a trend toward increased gastrointestinal toxicity with the brachytherapy boost, 8 vs. 3% (*p* = 0.12) (42). However, at the 6-years follow-up time point, health-related quality of life was similar between the two groups in most domains with the exception that physical and urinary function scales were lower in the LDR arm (43). Regardless, the increased toxicity observed in the combined EBRT plus LDR boost arm ASCENDE-RT highlights the importance of careful patient selection and diligent treatment planning as well as early intervention with symptom management as needed for these patients. A detailed analysis of the treatment related morbidity from the trial is available (42).

HDR brachytherapy may be a method to overcome the acute toxicities seen with LDR given the physical properties of this treatment modality. With an LDR implant, the radiation dose is delivered over a time period of months compared to minutes with HDR. For this reason, LDR is associated with a more prolonged recovery period. A prospective non-randomized comparison of quality of life after LDR vs. HDR boost (combined with 4.5 weeks of EBRT) showed a return to baseline IPSS at 6 months with LDR compared to only 12 weeks with HDR (44). Another early analysis of a randomized controlled trial of HDR vs. LDR in the monotherapy setting suggests improved quality of life, shorter return time to baseline urinary function, and lower rates of acute urinary symptoms with HDR monotherapy (45). While the previous studies show very favorable toxicity profiles with HDR brachytherapy compared to LDR, HDR has been associated with non-insignificant rates of urethral stricture. In a study by Bece et al. reported in 2015, various doses and fractionations of HDR boost (19.5 Gy/3fractions; 17 Gy/2 fractions; 18 Gy/2 fractions; and 19 Gy/2 fractions) in combination with EBRT were used and overall 3 and 6-years stricture incidence were 7.8 and 15.3%, respectively (46). The HDR boost fractionation scheme evolved during their study and the most recent fractionation used (19 Gy/2 fractions) resulted in the lowest three-year stricture rate of 3.0% (46). Yaxley et al. retrospectively analyzed a series of 507 men consecutively treated with EBRT plus HDR brachytherapy with a median follow-up of 10.3 years and found that rates of urethral stricture can be significantly reduced with careful attention to dose heterogeneity constraints, imaging prior to second HDR fraction to control for needle displacement, and tighter apical (inferior) PTV margins during the EBRT (47). Prior to implementation of these “stricture prevention measures,” the rate of stricture was 13.6% and this rate dramatically fell to 4.2% using these planning considerations (47).

In terms of long-term toxicity, an investigation using the Surveillance, Epidemiology, and End Results Medicare database (SEER) did not show a statistically significant difference in Grade 3 genitourinary adverse events between LDR and HDR (48). The results of the BrachyQOL randomized controlled trial (NCT01936883) are highly anticipated as they will shed more definitive light on both the acute and late GU/GI side-effect profiles between LDR and HDR in the boost setting (49).

## Declining use of brachytherapy boost

Despite potential improved outcomes with either LDR or HDR boost, the rates of brachytherapy boost utilization are declining (50, 51). Multiple reasons for the declining use of a boost have been reported including increase of prostatectomies for higher risk patients (52), increases in reimbursement for other EBRT techniques (53), decrease in brachytherapy training (54), and potential perception that brachytherapy is a procedure with excessive liability risk (51). In an analysis by Johnson et al. (19), the utilization of LDR brachytherapy boost dropped from ~29% in 2004 to 14% in 2012. Previous database-based studies also report the declining use of EBRT plus brachytherapy boost (52). The American Brachytherapy Society (ABS) has started a “300 in 10” initiative to increase the training of brachytherapists by assisting in the training of 30 oncologists per year over a 10-years period. Initiatives such as this are extremely important as a brachytherapy boost has the potential to improve prostate-specific survival outcomes when compared to EBRT alone.

## Future directions

While an optimal dose for LDR brachytherapy boost has been established, studies continue to determine the optimal HDR schedule and dose escalation continues to be investigated in the HDR setting. Also, limited data on prostate cancer specific survival outcomes between HDR and LDR exist. The British Columbia Cancer Agency is conducting a Phase III randomized controlled trial in patients with unfavorable intermediate risk and high-risk prostate cancer who will receive 46 Gy in 23 fractions of EBRT and then be randomized to either a LDR boost using ^125^I (115 Gy) or HDR boost using ^192^Ir (15Gy x 1). In addition to quality of life measures, a secondary outcome is PSA recurrence-free survival which will provide randomized head-to-head outcomes between LDR and HDR brachytherapy boost. Another unknown for both LDR and HDR boost in the high risk setting is defining the optimal planning target volume (PTV) to balance tumor coverage while minimizing toxicity. The differences in CTV to PTV expansion between RTOG 0019 and CALBG 99809 in the intermediate risk setting were 5 vs. 0 mm, respectively, and may have long term toxicity consequences. In high-risk prostate cancer, while the external beam target volumes should include any extracapsular extension and the at risk proximal seminal vesicles, some intuitions are including both the proximal seminal vesicles and extracapsular extension in the brachytherapy boost volume, but the clinical significance of such inclusion is unknown. Advanced computer planning and CT/MRI/Ultrasound-based planning with HDR brachytherapy may allow better and more reproducible coverage of the seminal vesicles and extracapsular extension compared to LDR given the inherent post-implant treatment planning capabilities with HDR. Additionally, as imaging technology continues to improve, small institutional trials are underway or have completed investigating focal brachytherapy boost to intraprostatic lesions using MRI-transrectal-ultrasound fusion (55). Lastly, SBRT continues to gain popularity given its shorter treatment course and less invasive nature, comparisons between SBRT and brachytherapy are emerging. Preliminary data from a reported literature search of 47 studies on PubMed and Embase (6 SBRT boost and 41 HDR boost), showed that a SBRT boost may be associated with higher acute Grade 2 genitourinary toxicity but lower late Grade 3 GU toxicity, and no difference was seen between the two by quality of life reports. Randomized trials between both LDR and HDR boost and SBRT boost are warranted and underway.

## Conclusions

Both HDR and LDR brachytherapy provide a method of biologically equivalent dose escalation in patients with high-risk prostate cancer who are undergoing definitive intent radiation therapy. In combination with EBRT, brachytherapy is a modality to deliver highly conformal dose escalation while drastically sparing the rectum and bladder compared to EBRT alone. Two randomized controlled trials have shown improved biochemical control with EBRT plus brachytherapy boost but neither demonstrated a statistical difference in overall survival (16, 25). The LDR boost arm in the ASCENDE-RT trial demonstrated a significant improvement in biochemical progression-free survival and long term survival outcomes are eagerly anticipated (9). Despite the improvements in biochemical control with brachytherapy boost, trends in the use of brachytherapy continue to decline nationally, possibly secondary to concerns of acute genitourinary toxicity with LDR. Initiatives to increase brachytherapy use are currently underway, and HDR brachytherapy may be an opportunity to improve toxicity profiles while exploiting the radiobiology of prostate cancer in the boost setting.

## Author Contributions

BF-V, HG, SP, BB, and JM: literature search, writing, and final approval.

### Conflict of Interest

The authors declare that the research was conducted in the absence of any commercial or financial relationships that could be construed as a potential conflict of interest.
