# Supplementary material for: A Brief Review of Low-Dose Rate (LDR) and High-Dose Rate (HDR) Brachytherapy Boost for High-Risk Prostate
Source: Front Oncol. 2019 Dec 10;9:1378. doi: 10.3389/fonc.2019.01378 (PMC6914687; doi:10.3389/fonc.2019.01378)
Supplement: Supplementary file 1 [file Data_Sheet_1.docx]

**Supplemental:**

**Methods:**

A PubMed (<https://www.ncbi.nlm.nih.gov/pubmed/>) literature search was performed using keyword criteria to include: high-risk prostate cancer, brachytherapy boost, high-dose rate (HDR) brachytherapy, low-dose rate (LDR) brachytherapy, and dose-escalated external beam radiation. Results were then prioritized based on level of evidence with randomized controlled trials receiving the highest priority followed by Phase II trials and multi-institutional trials and reports. Single institution reports and trials, and population/registry studies comparing HDR versus LDR in the high-risk setting were then given next priority. Lastly, studies with specific toxicity data were included for the review. In total, approximately 100 publications were reviewed and 50 included in the herein review paper.
